# Synthesis and Characterization of Solution-Processible Donor-Acceptor Electrochromic Conjugated Copolymers Based on Quinoxalino[2′,3′:9,10]phenanthro[4,5-abc]phenazine as the Acceptor Unit

**DOI:** 10.3390/polym15040940

**Published:** 2023-02-14

**Authors:** Zhen Xu, Bozhen Wang, Lingqian Kong, Jinsheng Zhao, Yuchang Du

**Affiliations:** 1 School of Chemistry and Chemical Engineering, Shandong University of Technology, Zibo 255049, China; 2 Dongchang College, Liaocheng University, Liaocheng 252059, China; 3 Shandong Key Laboratory of Chemical Energy Storage and Novel Cell Technology, Liaocheng University, Liaocheng 252059, China; 4 Key Laboratory of Jiangxi University for Applied Chemistry and Chemical Biology, College of Chemistry and Bioengineering, Yichun University, Yichun 336000, China

**Keywords:** conjugated copolymers, electrochromic, donor–acceptor, solution-processible

## Abstract

Donor-acceptor (D-A) type conjugated polymers are of high interest in the field of electrochromism. In this study, three novel conjugated copolymers (PBPE–1, PBPE-2 and PBPE-3) based on quinoxalino[2′,3′:9,10]phenanthro[4,5-abc]phenazine (A) as the acceptor unit and 4,8-bis((2-octyldodecyl)oxy)benzo[1,2-b:4,5-b′]dithiophene (D1) and 3,3-didecyl-3,4-dihydro-2*H*-thieno[3,4-b][1,4]dioxepine (ProDOT-decyl_2_, D2) as the donor units with different donor-to-acceptor ratios were successfully synthesized through Stille coupling polymerization. The polymers were then characterized by cyclic voltammetry (CV), fourier transform infrared (FT-IR) spectoscopy, X-ray photoelectron spectroscopy (XPS), spectroelectrochemistry, thermogravimetry (TG), electrochromic switching and colorimetry. Optical band gap values were calculated as 1.99 eV, 2.02 eV and 2.03 eV, respectively. The three copolymers have good solubility, distinct redox peaks, wide absorption spectra, good thermal stabilities, bright color changes and significant electrochromic switching properties. Compared to the other two copolymers, the PBPE-3 film exhibited high coloration efficiency values of 513 cm^2^·C^−1^ at 504 nm and 475 cm^2^·C^−1^ at 1500 nm. The films have the advantage of exhibiting cathodic and anodic coloration.

## 1. Introduction

In the past few decades, the field of conductive conjugated polymers has made great breakthroughs, and many organic optoelectronic materials [1,2,3] with excellent properties have been developed. Conductive polymers have been widely used in military camouflage, organic light-emitting diodes (OLED) [4,5,6], energy-saving materials, optical displays, organic thin-film transistors (OTFT) [7,8] and other fields [9] because of their excellent optoelectronic properties. Only donor-type polymers have shown potential in completing the RGB color spectrum [10,11,12,13,14]. Furthermore, the donor-acceptor (D-A) type of electrochromic (EC) material conjugated polymers [15,16,17,18,19] has the advantages of high environmental stability, rich color change, easy film formation, band gap adjustment, high coloring efficiency and contrast. The optical and electrochemical properties of the D-A type conjugated conductive polymer can be effectively adjusted by molecular structure design and modification [20,21]. Adjusting the band gap can effectively control the optical and electrochemical properties of the electrochromic polymer. Reynolds’ research group has done a lot of research by controlling the HOMO (highest occupied molecular orbital) and LUMO (lowest unoccupied molecular orbital) energy levels of the polymers to adjust the optical band gap [10,11,12]. In particular, the synthesis of low-band-gap polymers has been favored by researchers due to their low driving voltage, good p-type doping and n-type doping characteristics [22] and rich color rendering. Therefore, a large number of technical reports has been published on the design, synthesis and characterization of low-band-gap conjugated polymers [23,24,25,26,27,28]. The D-A type polymer containing an electron donor and an electron-accepting unit has a strong carrier-transporting ability, which is the most effective method to synthesize low-band-gap polymer materials. Therefore, the incorporation of alternating strong electron donor and electron acceptor units into a polymer is a common method for constructing low-band-gap system polymers because the donor-acceptor construction has higher HOMO and lower LUMO energy levels, which can effectively reduce the band gap for electron transition.

In this study, an indium tin oxide (ITO)-coated glass was used as an electrode to cover the electrochromic polymer film to promote ion migration [29]. We used benzodithiophene derivatives (BDT, D1) and ProDOT-decyl_2_ (D2) as the donor units. The D1 unit has a large planar conjugate structure, forming π-π stacking, which may result in high hole mobility and charge carrier transport rate [30,31,32]. At present, conjugated polymers based on benzodithiophene are mainly used in the construction of organic photovoltaic (OPV) [33,34], and there are relatively few reports on electrochromic properties [35]. In order to improve the solubility of polymer and achieve large-scale production and application of electrochromic materials, it is a useful and common method to incorporate long alkyl or alkoxy side chains into the backbone of the polymer molecular framework structure. In addition, Hou et al. found that the energy level and band gap of a polymer can be effectively adjusted by copolymerizing a benzodithiophene analog with different electron-rich or electron-deficient aromatic groups [30]. ProDOT exhibits excellent performance [36,37], with a high HOMO energy level and coloring efficiency, and the introduction of an alkyl side chain on the central carbon atom of the propylene bridge can also greatly increase the solubility of the copolymers. The optical and electrochemical properties of conjugated polymers are changed by the combination of electronic and spatial effects. Aromatic heterocyclic compounds containing C=N groups are often used as acceptors to construct polymers due to their electron-withdrawing ability and π-conjugated features. Structural modification of a traditional electron acceptor by hetero atom substitution or heterocyclic ringing can cause a change in energy band gap (E_g_), thereby adjusting the photoelectric properties of the conjugated polymers. In this paper, a novel compound, quinoxalino[2′,3’:9,10]phenanthro[4,5-abc]phenazine was used as the acceptor unit, which has a large planar structure and more active sites where the chemistry occurs, inducing hypercrossed polymers [38]. The acceptor unit can enhance the degree of conjugated electron delocalization and facilitates the absorption of photons.

On the foundation of above results, we used benzodithiophene derivatives (D1) and ProDOT-decyl_2_ (D2) as the donor units and a functionalized electron-deficient pragmatic molecule (A) as the acceptor unit. By changing the feed ratio of three monomers, three polymers were synthesized by Stille coupling reaction and named PBPE-1, PBPE-2 and PBPE-3, respectively. The structure, as well as electrochemical, kinetic, spectroelectrochemical and thermal stability, of the corresponding polymers, was researched in detail, as presented in the following sections.

## 2. Experimental

### 2.1. Materials

ProDOT-decyl_2_ was synthesized according to the reported procedure [39]. 1-Bromodecane, sodium hydride (NaH), lithium aluminum hydride (LiAlH_4_), tetrahydrofuran (THF), p-toluenesulfonic acid (PTSA, 98.5%), toluene, a 4A molecular sieve, trichloromethane, N-bromosuccinimide (NBS, 99.0%), sodium borohydride (NaBH_4_), ethyl alcohol, p-toluene sulfonic acid (PTSA), tetrabutylammonium hexafluorophosphate (TBAPF_6_, 98.0%), bis(triphenylphosphine)palladium(II)dichloride (Pb(PPh_3_)_2_Cl_2_) and anhydrous magnesium sulfate, were all purchased from commercial sources. 3,4-dimethoxythiophene and (4,8-Bis((2-octyldodecyl)oxy)benzo[1,2-b:4,5-b′]dithiophene-2,6-diyl)bis(triMethylstannane) (D1) were purchased from Derthon Optoelectronic Materials Science Technology Co., Ltd. (Shenzhen, China). The acceptor, 5,8,14,17-tetrabromoquinoxaline[2′,3′:9,10]phenanthro[4,5-abc] phenazine, was obtained from KWX Biopharmaceuticals, Inc. (Liaocheng, China).

Indium tin oxide (ITO)-coated glasses (sheet resistance: <10 Ω/sq) were purchased from Zhuhai Kaivo Optoelectronic Technology Co., Ltd. (Zhuhai, China), and washed with anhydrous ethanol, acetone and distilled water in an ultrasonic bath before use. Reagents were all of analytical grade and used as received unless otherwise stated.

### 2.2. Instrumentation

^1^H and ^13^C NMR spectra were measured by a Varian AMX 400 NMR spectrometer (Varian, Palo Alto, CA, USA) with tetramethylsilane as internal standard and CDCl_3_ as solvent. Cyclic voltammetry (CV) tests were carried out on a CHI760D electrochemical workstation (Chen Hua Instrument Co., Ltd., Shanghai, China), and in the tests, a custom-made three-electrode cell system was used, with ITO conductive glass (coated with polymer films obtained by spray coating method), silver wire and platinum wire used as working electrode, reference electrode and counter electrode, respectively. Switching kinetics, spectroelectrochemistry and colorimetry of the polymers were obtained using a Varian Cary 5000 UV-Vis-NIR spectrophotometer (Agilent Technologies Ltd., Mulgrave, Australia) in conjunction with a CHI760D electrochemical workstation. Fourier transform infrared spectroscopy (FT-IR) was conducted with a Nicolet 6700 FT-IR spectrometer (Thermo Nicolet Corporation, Fitchburg, WI, USA). X-ray photoelectron spectroscopy (XPS) analyses were measured on a thermo Escalab Xi+ (Thermo Fisher Scientific, Waltham, MA, USA) with a monochromated Al X-ray resource. Thermogravimetric analysis (TGA, NETZSCH Scientific Instruments Trading Ltd., Selb, Germany) was performed to record the thermal stability. Experiments were carried out in an acetonitrile (ACN) medium containing 0.2 M TBAPF_6_ as a supporting electrolyte. The color changes of the polymer films were recorded in real time with a camera.

### 2.3. Synthesis of Monomers and Polymers

Detailed synthesis routes of the donor ProDOT-decyl_2_ are described in the Supplementary Materials (Figure S1). ^1^H NMR and ^13^C NMR spectra of the intermediate and the target donor products are also shown in Figures S2 and S3.

**Figure 1:**
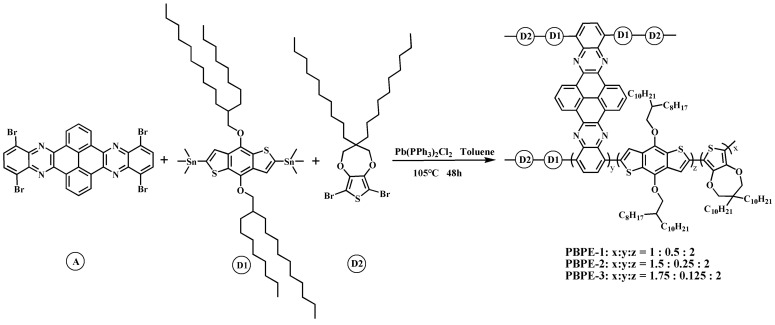
Synthetic routes of PBPE-1, PBPE-2 and PBPE-3.

Three D-A type polymers—PBPE-1, PBPE-2 and PBPE-3—were synthesized by Stille coupling reaction. The synthetic route is shown in Figure 1. The molar ratio of the three monomers used in the synthesis of the PBPE-1 polymer was D1:D2:A = 2:1:0.5. The detailed process is as follows: D1 (499.7 mg, 0.4500 mmol), D2 (133.2 mg, 0.225 mmol), A (80.7 mg, 0.1125 mmol), Pb(PPh_3_)_2_Cl_2_ catalyst (18.0 mg, 0.0256 mmol) and 60 mL of toluene were separately added to a 250 mL round-bottom flask. The air in the reaction apparatus was evacuated under a reduced pressure device and replaced with argon three times. The reaction was carried out in an argon atmosphere for 48 h, and the reaction temperature was 105 °C. After completion of the reaction, the solvent was removed by rotary evaporation. The solid sample was purified by Soxhlet extractor and extracted by n-hexane, methanol and acetone for 24 h. Finally, the PBPE-1 solid polymer was dried in an oven with a yield of 81.4%. ^1^HNMR data of polymer PBPE-1 (Figure S4a) were as follows: ^1^HNMR (CDCl_3_, 400 MHz, ppm): 7.45 (s, 21H), 7.19–7.05 (m, 38H), 4.23–3.88 (m, 8H), 1.90 (s, 35H), 1.46–1.04 (d, 240H), 1.01–0.78 (t, 79H), 0.68 (s, 44H); GPC: M_w_ = 15.50 kDa; M_n_ = 14.90 kDa; PDI = 1.04.

The molar ratio of the three monomers used in the synthesis of the PBPE-2 polymer was D1: D2: A = 2: 1.5: 0.25. D1 (499.7 mg, 0.4500 mmol), D2 (199.9 mg, 0.3375 mmol), A (40.4 mg, 0.0563 mmol), Pb(PPh_3_)_2_Cl_2_ catalyst (18.0 mg, 0.0256 mmol) and 60 mL of toluene were added into a 250 mL round-bottom flask in turn. Other reaction conditions and post-treatment methods were consistent with those of PBPE-1, with a yield of 78.4%. ^1^HNMR data of polymer PBPE-2 (Figure S4b) were as follows: ^1^HNMR (CDCl_3_, 400 MHz, ppm): 7.46 (s, 15H), 7.21–7.07 (d, 30H), 4.13–3.93 (d, 8H), 1.90 (s, 15H), 1.47–1.03 (d, 282H), 0.91 (t, 80H); GPC: M_w_ = 14.68 kDa; M_n_ = 13.60 kDa; PDI = 1.08.

The molar ratio of the three monomers used in the synthesis of the PBPE-3 polymer was D1:D2:A = 2:1.75:0.125. D1 (499.7 mg, 0.4500 mmol), D2 (233.2 mg, 0.3938 mmol), A (20.2 mg, 0.0281 mmol), Pb(PPh_3_)_2_Cl_2_ catalyst (18.0 mg, 0.0256 mmol) and 60 mL of toluene were added into a 250 mL round-bottom flask in turn. Other reaction conditions and post-treatment methods were also consistent with those of PBPE-1, with a yield of 77.6%.^1^HNMR data of polymer PBPE-3 (Figure S4c) were as follows: ^1^H NMR (CDCl_3_, 400 MHz, ppm): 7.38 (s, 13H), 7.21–6.95 (m, 18H), 4.13–3.93 (m, 8H), 1.90 (s, 20H), 1.45–1.15 (m, 156H), 1.11–0.71 (m, 66H); GPC: M_w_ = 17.52 kDa; M_n_ = 16.07 kDa; PDI = 1.09.

### 2.4. Film Preparation

The three D-A type polymers were dissolved in chloroform to give a concentration of 5 mg/mL. The obtained solutions were sprayed on the ITO glass using an art spray gun, and the effective area of the polymer film was 3 cm^2^ (3 cm × 1 cm). ITO-coated glass slides should be ultrasonically cleaned with acetone, ethanol and deionized water for 15 min and dried by nitrogen blowing before use.

## 3. Results and Discussions

### 3.1. FT-IR Spectra

**Figure 2:**
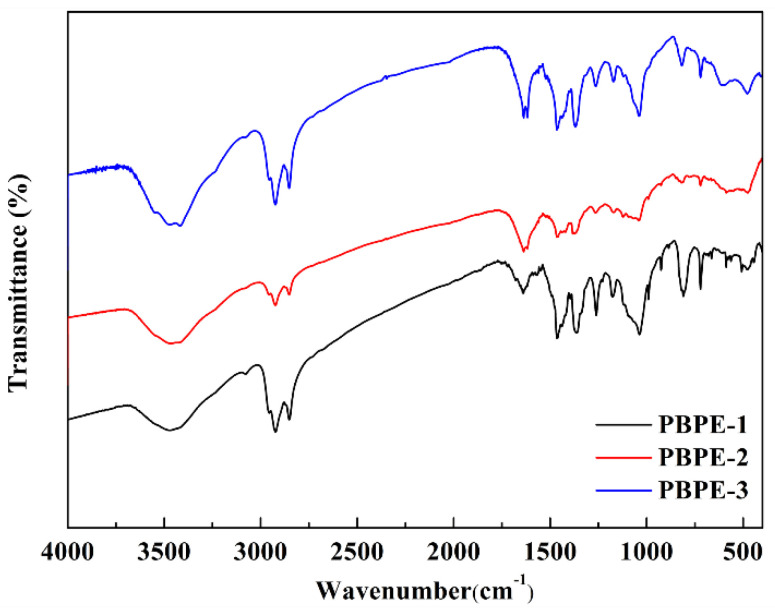
FT-IR spectra of PBPE-1, PBPE-2 and PBPE-3.

In order to further verify the structure of the polymers, we tested the FT-IR spectra of three polymers; the results are shown in Figure 2. Based on the infrared spectra of the three polymers, it is apparent that their absorption peak positions are very close to each other but differing in peak intensity. This is because the three types of comonomers are the same, but the composition ratios are different. Taking PBPE-3 as an example, the absorption peak at 3417 cm^−1^ is due to the stretching vibration of unsaturated C-H bonds on benzene rings or thiophene rings. The absorption peaks at 2923 cm^−1^ and 2852 cm^−1^ are due to the stretching vibration of the C-H bond in the saturated methyl group and methylene group of the side chain alkyl chain or the alkoxy chain of the donor units. The absorption peaks at 1637 cm^−1^, 1618 cm^−1^ and 1465 cm^−1^ are caused by the vibration of the skeleton of the tetraphenylquinoxaline phenanthroline and the benzene ring in the D1 unit. The absorption peaks at 1369 cm^−1^, 1263 cm^−1^ and 1038 cm^−1^ are attributed to the stretching vibration of C=N, C-S and C-O bonds, respectively. The bending vibration of the C-H bond on the benzene ring in tetrabromoquinoxaline phenanthroline or the D1 unit resulted in an absorption peak in the fingerprint region of the infrared spectra at 817 cm^−1^, 720 cm^−1^ and 478 cm^−1^. Testing confirmed that PBPE-1, PBPE-2 and PBPE-3 all contained characteristic functional groups of monomer 1, monomer 2 and monomer 3.

### 3.2. XPS Investigation of the Copolymers

**Figure 3:**
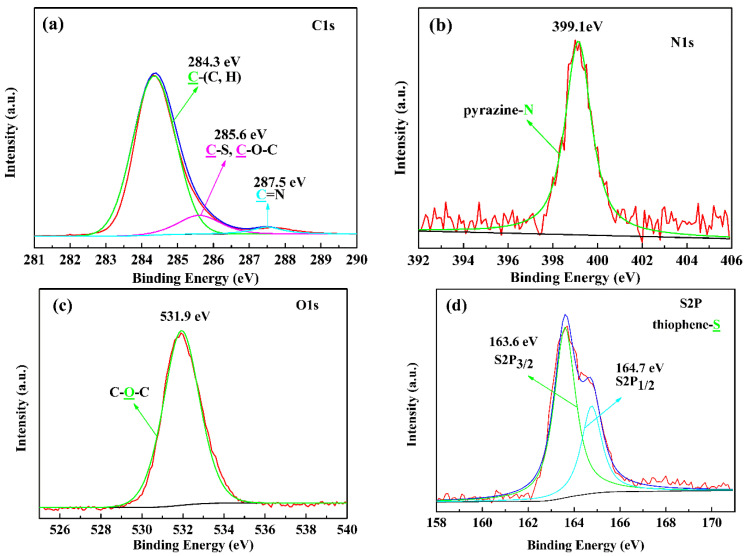
XPS spectra of polymer PBPE-1: (**a**) C1s, (**b**) N1s, (**c**) O1s and (**d**) S2p. The colored arrows point to the corresponding elemental valence states and their peaks.

In order to obtain the elemental composition information, we performed X-ray photoelectron spectroscopy (XPS) tests on three polymer films. The test results show that there are four characteristic elements including C, N, O and S in the three D-A type polymers, as shown in Figure 3. Taking PBPE-1 as an example, the C (1s) peak at 284.3 eV demonstrates the presence of sp^3^ hybridized carbon atoms derived from the alkyl or alkoxy chains in D1 and D2; the peaks at 285.6 eV are ascribed to the binding energy of C-O-C and C-S bands in D1 and D2; and the binding energy peak at 287.5 eV is attributed to the C=N band in the A group, indicating the coexistence of three structural composition participants. The N (1s) peak with a binding energy of 399.1 eV can be ascribed to pyrazine-N [40] in the A unit. The O (1s) peak at a binding energy of 531.9 eV is attributed to the presence of C-O-C ether linkage [41]. The presence of C-S bands in thiophene rings was confirmed by the binding energy peak at 163.6 eV (S 2p_3/2_) and the peak at 164.7 eV (S 2p_1/2_). Similar results were obtained for the PBPE-2 and PBPE-3 polymers; the XPS spectra are presented in Figures S5 and S6. XPS data further indicate that D-A type random copolymers containing three monomer structures were successfully prepared.

### 3.3. Electrochemical Characterization

**Figure 4:**
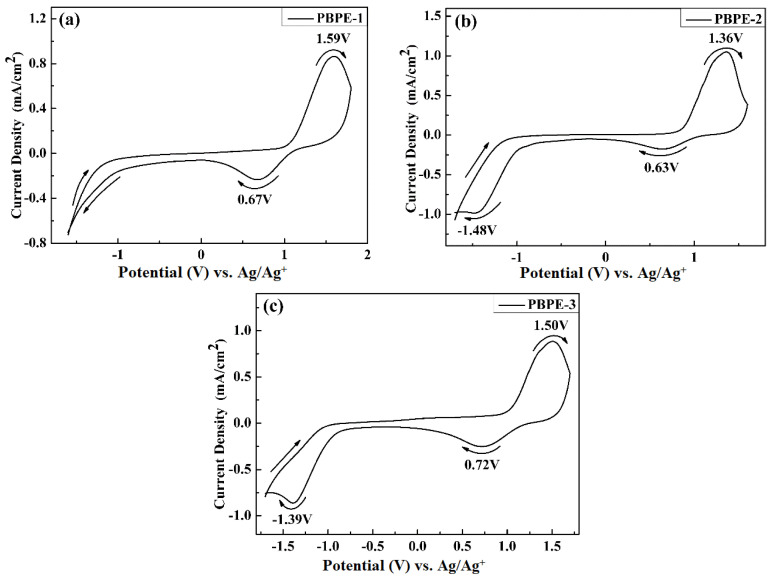
CV curves of copolymer films PBPE-1 (**a**), PBPE-2 (**b**) and PBPE-3 (**c**) on the spray-coated indium tin oxide (ITO) substrates in 0.2 M TBAPF_6_/ACN electrolyte solution at a scan rate of 100 mV/s.

The electrochemical tests were carried out on an electrochemical cell consisting of a polymer-coated ITO/glass (3.0 cm × 1 cm) as the working electrode, Pt wire as the counter electrode and an Ag wire as the pseudo-reference electrode. The electrochemical properties of the polymers were tested and studied using a CHI 760 electrochemical workstation in a 0.2 M TBAPF_6_/ACN electrolyte solution. The voltage scanning ranges of PBPE-1, PBPE-2 and PBPE-3 were −1.6~2 V, −1.6~1.8 V and −1.8~1.7 V, respectively, and the scanning rate was 100 mV/s. The cyclic voltammetry curves are shown in Figure 4. In the cyclic voltammetry (CV) cycle, the three D-A type polymers were oxidized slowly with increasing applied voltage, with the oxidation peaks of PBPE-1, PBPE-2 and PBPE-3 located at 1.59 V, 1.36 V and 1.50 V, respectively, due to the generation of polarons during the p-type doping process. In the dedoping process, the reduction peaks of the three polymers appeared at 0.67 V, 0.63 V and 0.72 V, respectively. All three polymers have a distinct pair of redox peaks and approximately the same peak intensity, indicating their good doping and dedoping capabilities.

In addition, based on the cyclic voltammetry curve, the initial oxidation potential (*E*_onset_) of PBPE-1, PBPE-2 and PBPE-3 were calculated be 1.07 V, 0.86 V and 1.03 V, respectively. Related literature indicates that the addition of donor units to the polymer of the D-A structure advantageously reduces the potential required to form free-radical cations to make the polymer more susceptible to oxidation. However, PBPE-2 had a moderate donor-acceptor ratio and a relatively low oxidation potential, which may be due to the balance between steric and electronic effects, resulting in the relatively fast electron transfer of the polymer. Compared to the p-type doping and de-doping process, the n-type doping process requires more stringent conditions and is extremely unstable in an electrolyte solution with water or oxygen, so that no significant n-type doping process occurs in the negative-potential portion.

### 3.4. Optical Properties of Polymer Films and Solutions

**Figure 5:**
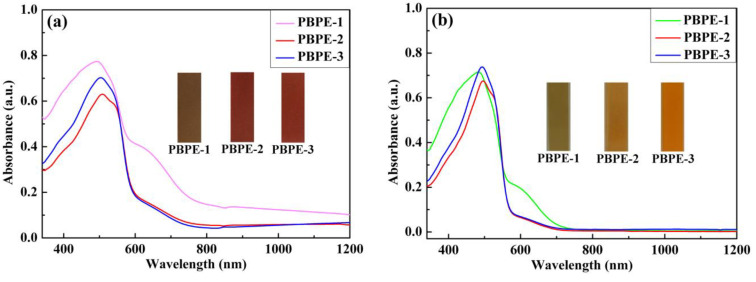
Absorption spectra and color photos of three polymer films (**a**) and solutions (**b**).

The optical properties of three D-A type polymer films and solutions were measured by a UV-Vis-NIR spectrophotometer, as shown in Figure 5. The UV-Vis spectra of the polymer films and solutions showed main broad absorption bands in the range of about 400 nm to 600 nm, with the maximum absorption peaks (λ_max_) of the polymers in solutions located at 481 nm, 496 nm and 493 nm, and λ_max_ in films at 492 nm, 508 nm and 503 nm, for PBPE-1, PBPE-2 and PBPE-3, respectively. The maximum absorption peaks in films showed a weak bathochromic shift compared with those in solutions due to the accumulation of π-π stacking of the polymer films, resulting in reduced energy absorption. Compared with PBPE-1, the maximum absorption peaks of PBPE-2 and PBPE-3 polymer films or the polymer solutions showed a red-shift phenomenon, and the red shift of PBPE-2 was more obvious. This may be because an appropriate increase in donor ratio results in a more planar and rigid backbone of the polymer, which is conducive to electronic transition. Corresponding to the spectral absorption, the PBPE-1 film appeared brown with a relatively broad absorption peak, and the PBPE-2 and PBPE-3 films exhibited a similar magenta color due to their similar and adjacent absorption peaks. Furthermore, the absorption humps in the range of 650 nm can be ascribed to D-A charge transfer interactions. The optical and electrochemical data of three polymers derived from CV curves and UV-Vis spectra are listed in Table 1.

### 3.5. Spectroelectrochemical Properties

**Figure 6:**
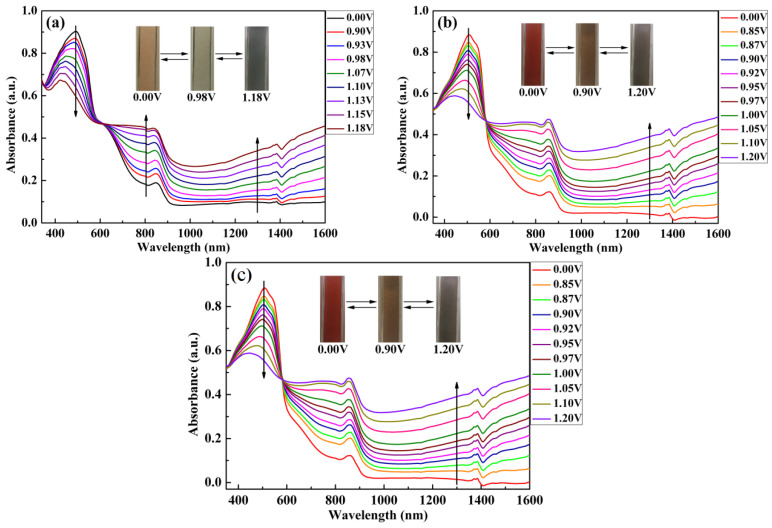
Spectroelectrochemical spectra of PBPE-1 (**a**), PBPE-2 (**b**), PBPE-3 (**c**) and corresponding color changes under different voltages.

Spectroelectrochemical performance was tested by stepping up the voltage [43], and the color changes of the three polymer films from the neutral state to the oxidation state were recorded, as shown in Figure 6. For PBPE-1, when the voltage was 0 V, the polymer had a distinct absorption peak at 492 nm due to the π-π* electronic transition [44]. With the increase in voltage, the absorption peak intensity in the visible region was gradually reduced. A new absorption peak also appeared at 803 nm, as well as a new wide absorption band in the near-infrared region, due to the generation of polarons and bipolarons [39]. When the voltage was increased to 0.98 V, the color of the polymer film changed significantly from a light yellow–brown to a light gray; when the voltage was increased to 1.18 V, the polymer film was completely oxidized and appeared cyan gray. The maximum absorption peak of PBPE-2 was located at 508 nm in the neutral state. As the oxidation voltage increased, similar changes occurred in the spectral absorption curves compared to PBPE-1. When the voltage reached 0.9V, the polymer film changed from crimson to straw in color, and the completely oxidized polymer film became dark gray. PBPE-3 film is very similar to PBPE-2 in terms of its curve and color changes. In addition, polymers PBPE-1, PBPE-2 and PBPE-3 have equal extinction points at 610 nm, 583 nm and 573 nm, which means that the polymers can easily convert between the doped state and the dedoped state.

### 3.6. Electrochromic Switching Studies

**Figure 7:**
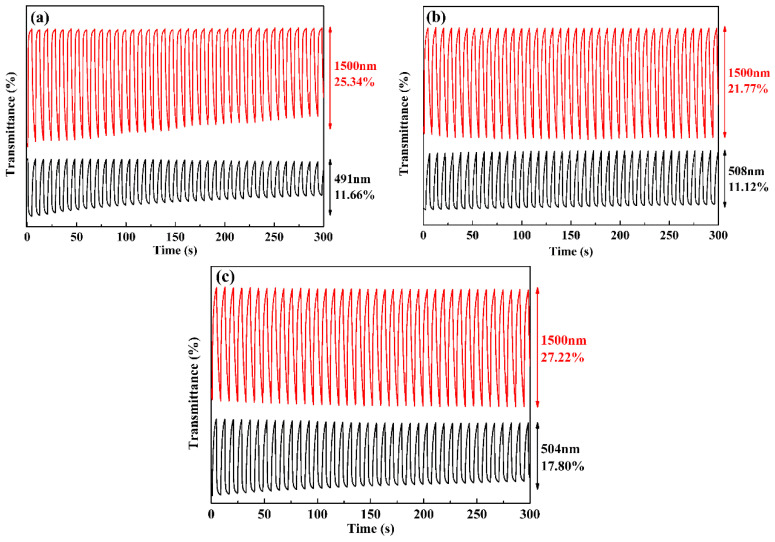
Square-wave potential step absorptiometry of PBPE-1 (**a**), PBPE-2 (**b**) and PBPE-3 (**c**) films monitored at various wavelengths in the visible and near-infrared regions.

In order to more fully evaluate the electrochromic properties of the polymers, dynamic tests of the polymers were carried out via cyclic square-wave voltammogram steps using a Varian Cary 5000 UV-Vis-NIR spectrophotometer in conjunction with a CHI 760D electrochemical workstation, and the critical aspects including optical contrast ratio (∆*T*%), response time (switch speed, t_95%_) and coloration efficiency (*η*) were obtained. Figure 7 shows the changes in optical transmittance curves of the three polymers in the visible or near-infrared region obtained by repeated scanning of the applied potential between the neutral and the oxidation state under a pulse duration of 4 s.

The optical contrast ratios of PBPE-1 film were calculated to be 11.66% at 491 nm in the visible region and 25.34% at 1500 nm in the near-infrared region. PBPE-2 had an optical contrast of 11.12% at 508 nm and 21.77% at 1500 nm. This is a slightly better parameter than that for PBPE-3, which was 17.80% at 504 nm and 27.22% at 1500 nm. In comparative experimental tests, the change in optical transmittance was not much different, but the stabilities of PBPE-2 and PBPE-3 were better than that of PBPE-1, as reflected by the decay of transmittance. In addition, the response times of three polymers at the wavelength of visible light and near-infrared light were calculated according to the times required for 95% complete oxidation and 95% complete reduction, which are listed in Table 2.

Another important factor, coloration efficiency (*η*), refers to the ratio of optical density (Δ*OD*) to the amount of charge (Δ*Q*) injected/ejected per unit area on the electrode, which was calculated according to Equations (1) and (2):

*η* = Δ*OD*/Δ*Q* (1)

Δ*OD* = log(*T*_b_/*T*_c_) (2)

where Δ*Q* is the charge inserted per unit area (*Q*/*A*), *Q* is the total amount of charge obtained by multistep potential integration, A is the effective area of the film on the 3 cm^2^ (3 cm × 1 cm) ITO conductive glass, *T*_b_ represents the maximum value of the polymer in the neutral state and *T*_c_ is the maximum value of the transmittance in the oxidation state [42,45]. As evidenced by the coloration efficiency data presented in Table 2, with the increase in donor ratio, the coloring efficiency of polymers showed a significant increase, especially for PBPE-3. Such a high coloring efficiency is suitable for research and the development of displays.

**Figure 8:**
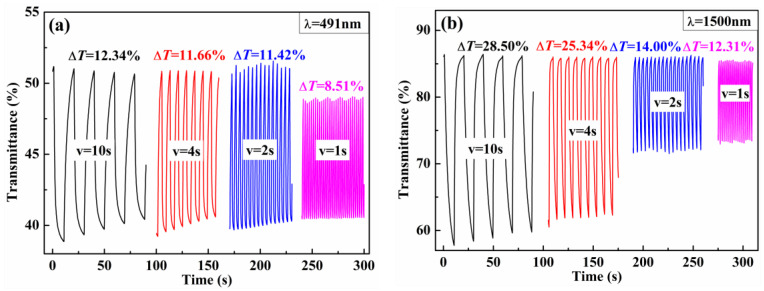
Electrochromic switching of PBPE-1 at 491 nm and 1500 nm with intervals of 10 s, 4 s, 2 s and 1 s.

The cyclic square-wave voltammogram steps at different wavelengths of the polymers with 10 s, 4 s, 2 s and 1 s pulse times were tested in order to study the stability of the polymers and the influence of pulse time on optical contrast, as shown in Figure 8. For PBPE-1, at 491 nm, the ΔT% value was 12.34% with a pulse time of 10 s. When the pulse time was reduced to 4 s, 2 s and 1 s, the ΔT% value dropped to 11.66%, 11.42% and 8.51%, respectively. From 10 s to 1 s, the ΔT% value at 1500 nm dropped from 28.5% to 12.31%. Decreasing the step time intervals from 10 s to 1 s resulted in a reduction in the EC contrasts of PBPE-2 from 16.67% to 5.45% at 508 nm and from 34.07% to 9.83% at 1500 nm (Figure S7). The degrees of reduction were calculated to be 12.94% and 30.9% for PBPE-3 at 504 nm and 1500 nm, respectively (Figure S8). As can be seen from the above data, the optical contrast value is closely related to the length of residence time. Meanwhile, in general, the faster the response speed, the less the contrast change is affected by the residence time.

### 3.7. Colorimetry

**Figure 9:**
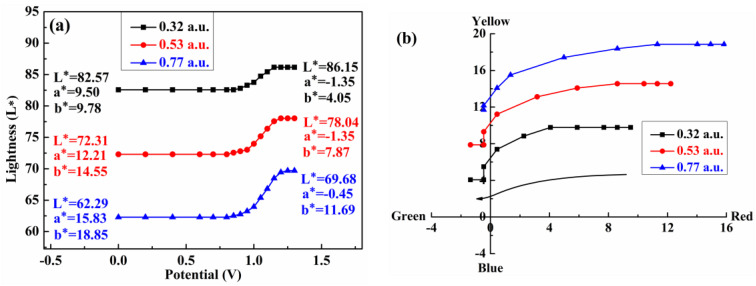
(**a**) Lightness (L*) and (**b**) colorimetry values (a* b*) for the PBPE-1 films at three different optical densities as a function of applied potential.

Colorimetric tests of the polymers were carried out using the CIE1976 L*a*b* color space to study the color changes of the polymer films. Figure 9 shows the brightness (L*) and color (a*, b*) changes of polymer films of different thicknesses achieved by gradually increasing the applied voltages. In this color space, L* indicates brightness (a value of 0 indicates black, and a value of 100 indicates white-positive) and a* and b* express red and yellow, respectively, while the negative values represent blue and green, respectively.

Three PBPE-1 polymers were selected with thicknesses of 0.32 au, 0.53 au and 0.77 au. In the neutral state, their brightness values were 82.57, 72.31 and 62.29, respectively. Before 0.75 V, the brightness of the films did not change significantly. As the voltage continued to increase, the polymer was oxidized, and the brightness was increased to 86.15, 78.04 and 69.68, respectively. As shown in Figure 9b, the (a*, b*) values of the polymer films with thicknesses of 0.32 a.u., 0.53 a.u. and 0.77 a.u. in a neutral state were (9.50, 9.78), (12.21, 14.55) and (15.83, 18.85), respectively; after the films were completely oxidized, the (a*, b*) values shifted to (−1.35, 4.05), (−1.35, 7.87) and (−0.45, 11.69), respectively. The polymer films with different thicknesses exhibited significant numerical changes in the L*, a* and b* values from the neutral state to the oxidation state, indicating that the transparence and the color of the polymer films underwent significant changes. The chromaticity test data of PBPE-2 and PBPE-3 are shown in Figures S9 and S10, respectively.

### 3.8. Thermogravimetric Analysis

**Figure 10:**
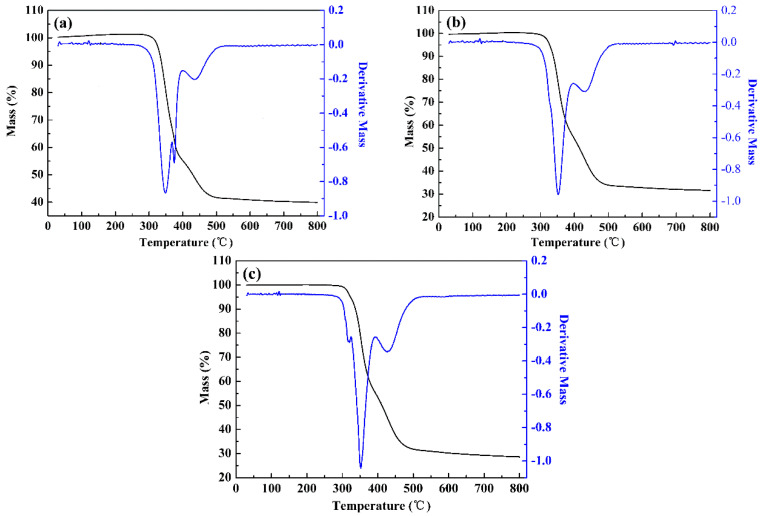
TG (black) and DTG (blue) curves of PBPE-1 (**a**), PBPE-2 (**b**) and PBPE-3 (**c**).

The thermal characteristics of three D-A type conjugated copolymers were analyzed by thermogravimetric analysis (TGA) and derivative thermogravimetry (DTG). When the thermal energy applied to the material exceeded the chemical bond energy of the molecule, the structure of the polymer was destroyed, which led to the decomposition of the polymer. Figure 10 shows the TG and DTG curves for the three polymers obtained in thermogravimetric tests. The characteristic decomposition temperatures of three polymers were obtained by tangent to the TG curve, and the extrapolation onset temperatures were 323 °C, 326 °C and 324 °C, respectively; it can be seen that all three polymers have relatively high characteristic decomposition temperatures. In addition, according to the peak value of the DTG curve, the fastest decomposition temperatures of the three polymers were 348 °C, 351 °C and 352 °C, respectively. In the TG curves, when the final temperature rose to 800 °C, the coke yields (% char) of the three polymers were 40%, 31% and 28%, respectively, which may be due to the presence of a large number of aromatic groups in the main chain of the polymer. Analysis of the above data shows that the three polymers have the ability to withstand high temperature with good thermal stability, indicating broad prospects for the application of these polymers.

## 4. Conclusions

In summary, three D-A type conjugated copolymers (PBPE-1, PBPE-2 and PBPE-3) based on ProDOT-decyl_2_ and benzodithiophene as the donor units and a new bi-quinoxaline compound, 5,8,14,17-tetrabromoquinoxaline [2′,3′:9,10] phenanthrene [4,5-abc] phenozine, as the acceptor unit, were successfully synthesized via Stille cross-coupling polymerization. The structure of the polymers was proven by NMR, FT-IR and X-ray photoelectron spectroscopy (XPS). They were characterized by different optical and electrochemical testing methods, the results of which showed that all three polymers have good electrochemical and electrochromic behaviors, showing reversibly rich and bright color changes between neutral and oxidized states. PBPE-2 had the lowest initial oxidation potential of 0.86 V, and PBPE-3 had a high coloring efficiency of up to 513 cm^2^·C^−1^. All three polymers showed definite optical contrast, cycling stability, fast response time and high thermodynamic stability. Consequently, our results indicate that cross-linked D-A type and soluble conjugated polymers can be prepared by adopting a multi-functionalized unit (four) with a bisquinoxaline structure as the acceptor unit and alkylated BDT and ProDOT unit as the donor units. The photoelectric properties of the resultant polymers, including the light absorption range and electrochromic kinetic properties, can be modulated by changing the feed ratios of the donor units to the acceptor unit in a synthetic procedure. More efforts should be devoted to the development of this type of polymer due to their expectant properties, especially in the electrochromic field.

## Figures and Tables

**Table 1:** Parameters of the optical and electrochemical properties of three polymers.

Polymer Film	*λ_max_* (nm)	*λ_onset_* (nm)	*E_onset,ox_* (V)	*E_onset,red_* (V)	HOMO (eV) ^a^	LUMO (eV) ^b^	*E_g,ec_* (eV) ^c^	*E_g,op_* (eV) ^d^
PBPE-1	492	624	1.07	−0.98	−5.32	−3.27	2.05	1.99
PBPE-2	508	615	0.86	−1.00	−5.11	−3.25	1.86	2.02
PBPE-3	503	610	1.03	−0.96	−5.28	−3.29	1.99	2.03

^a^ Fc/Fc+ potential was also measured to calibrate the pseudo-Ag wire electrode (0.55 V vs. Ag wire electrode). *E*_HOMO_ (eV) = −4.8 − (*E*_onset,ox_ − 0.55). ^b^ *E*_LUMO_(eV) = −4.8 − (*E*_onset,red_ − 0.55). ^c^ *E*_g,ec_ (eV)= *E*_LUMO_ − *E*_HOMO_. ^d^ *E*_g,op_ (eV)= 1241/λ_onset_ [42].

**Table 2:** Optical contrasts (∆T%), coloration efficiencies (CE) and response times (t_95%_) of PBPE-1, PBPE-2 and PBPE-3.

Polymer	Optical Contrast (∆T %)	CE (cm^2^·C^−1^)	Response Time (s)	
			t_b_ (Time for Oxidation)	t_c_ (Time for Reduction)
PBPE-1	11.66% (491 nm)	145	2.89	1.52
	25.34% (1300 nm)	268	1.22	3.21
PBPE-2	11.12% (508 nm)	229	3.43	1.33
	21.77% (1500 nm)	274	2.36	3.38
PBPE-3	17.80% (504 nm)	513	3.00	1.32
	27.22% (1500 nm)	475	2.92	3.41

## Data Availability

All related additional data are available in the Supplementary Materials.

## Supplementary Materials

The following supporting information can be downloaded at: https://www.mdpi.com/article/10.3390/polym15040940/s1, Figure S1: synthesis route of the donor 2; Figure S2: ^1^H NMR (a) and ^13^C NMR (b) spectra of 3,3-didecyl-3,4-dihydro-2H-thieno[3,4-b][1,4]dioxepine, ‘x’ represents the CDCl_3_ solvent peak and ‘y’ represents the water peak; Figure S3: ^1^H NMR (a) and ^13^C NMR (b) spectra of 6,8-dibromo-3,3-didecyl-3,4-dihydro-2H-thieno[3,4-b][1,4]dioxepine, ‘x’ represents the CDCl_3_ solvent peak and ‘y’ represents the water peak; Figure S4: ^1^H NMR spectra of PTPE-1(a), PTPE-2(b), PTPE-3(c); Figure S5: XPS spectra of polymer PBPE-2: (a) C1s, (b) N1s, (c) O1s and (d) S2p; Figure S6: XPS spectra of polymer PBPE-3: (a) C1s, (b) N1s, (c) O1s and (d) S2p; Figure S7: Electrochromic switching of PBPE-2 at 508 nm and 1500 nm with intervals of 10 s, 4 s, 2 s and 1 s; Figure S8: Electrochromic switching of PBPE-3 at 504 nm and 1500 nm with intervals of 10 s, 4 s, 2 s and 1 s; Figure S9: (a) Lightness (L*) and (b) colorimetry values (a* b*) for the PBPE-2 films in three different optical densities as a function of applied potential; Figure S10: (a) Lightness (L*) and (b) colorimetry values (a* b*) for the PBPE-3 films in three different optical densities as a function of applied potential.
