# Supplementary material for: Synthesis and Characterization of Solution-Processible Donor-Acceptor Electrochromic Conjugated Copolymers Based on Quinoxalino[2′,3′:9,10]phenanthro[4,5-abc]phenazine as the Acceptor Unit
Source: Polymers (Basel). 2023 Feb 14;15(4):940. doi: 10.3390/polym15040940 (PMC9965564; doi:10.3390/polym15040940)
Supplement: Supplementary file 1 [file polymers-15-00940-s001.zip › polymers-2133497-supplementary.pdf]

Synthesis and characterization of solution-processible donor-acceptor electrochromic conjugated copolymers based on quinoxalino[2′,3′:9,10]phenanthro[4,5-abc]phenazine as the acceptor unit

Zhen Xu[1], Bozhen Wang[1], Lingqian Kong[2], Jinsheng Zhao[3,*], Yuchang Du[4,*]

[1] School of Chemistry and Chemical Engineering, Shandong University of Technology, Zibo 255049, China

[2] Dongchang College, Liaocheng University, Liaocheng, 252059, China

[3] Shandong Key Laboratory of Chemical Energy Storage and Novel Cell Technology, Liaocheng University, Liaocheng, 252059, China

[4] Key Laboratory of Jiangxi University for Applied Chemistry and Chemical Biology, College of Chemistry and Bioengineering, Yichun University, Yichun, 336000, China

*Corresponding Author: j.s.zhao@163.com (J. S. Zhao); yuchdu@126.com (Y. C. Du)

1. Synthesis for monomers and polymers

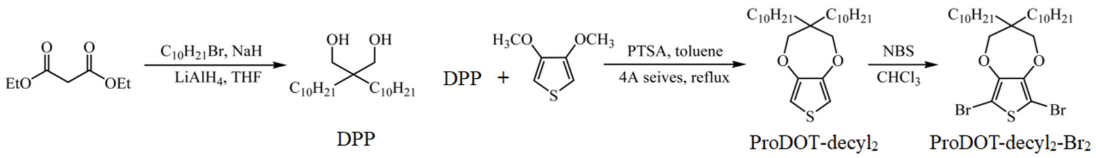

Figure. S1 synthesis route of the donor 2

200 mL of anhydrous tetrahydrofuran, 35 mmol of 1-bromodecane, and 35 mmol of NaH were separately added to a 500 mL three-neck roundbottom flask. 11.5 mmol of diethyl malonate was added dropwise to the solution mixture at 0°C. The reaction was carried out in argon atmosphere for 24 hours under reflux. When the flask was cooled to 0°C, the mixture was poured into brine (200 mL) and extracted twice with ether. Then the solvent was removed by rotary evaporation to give crude alkylated diethyl malonate (AMD) for the next step. In a 500 mL three-neck round-bottom flask, 11.3 mmol AMD was added dropwise to 200 mL of diethyl ether containing 17 mmol of $LiAlH_4$ powder at 0°C. The mixture was stirred under argon for 20 h at room temperature. The reaction product was filtered and vacuum distilled to provide 2,2-didodecylpropane-1,3-diol (DDP). 200 mL of toluene, 40 mmol of DDP, 20 mmol of 3,4-dimethoxythiophene, and 2.0 mmol of p-toluenesulfonic acid (PTSA) were separately added to a 500 mL roundbottom flask equipped with a Soxhlet extractor with 4A molecular sieves in a cellulose thimble. The mixture was stirred under argon for 24 h under reflux. When the flask was cooled to room temperature, the mixture was washed once with water. Then the solvent was removed by rotary evaporation. The crude mixture was chromatographed on silica gel by eluting with hexane: dichloromethane (3:2, v/v) to give ProDOT-decyl$_2$ as yellow oil (68 %). ^{1}H NMR ($CDCl_3$, 400 MHz, δ ppm): 6.42 (s, 2H), 3.84 (s, 4H), 1.26 (m, 36H), 0.88 (t, 6H). ^{13}C NMR ($CDCl_3$, 101 MHz, ppm): 149.69, 104.63, 77.53, 77.00, 76.68, 43.72, 29.63, 29.33, 22.68, 14.13 (see in Figure. S2).

150 mL of chloroform and 4.2 mmol of ProDOT-decyl$_2$ were added into a two-neck 250 mL round-bottom flask. Then 12.6 mmol of N-bromosuccinimde (NBS) was added, and the solution was stirred under argon for 20 h. After completion of the reaction, the solvent was removed by rotary evaporation. The crude residue was purified by column chromatography (hexane: dichloromethane (5:1, v/v)) to give the product ProDOT-decyl$_2$-Br$_2$(Yield, 89 %). ^{1}H NMR ($CDCl_3$, 400 MHz, δ ppm): 3.90 (s, 4H), 1.26 (m, 36H), 0.88 (t, 6H). ^{13}C NMR ($CDCl_3$, 101 MHz, ppm): 147.14, 90.63, 77.98, 77.00, 76.68, 43.99, 31.65, 29.72, 22.69, 14.10 (see in Figure. S3).

2. NMR spectra of the monomers and polymers

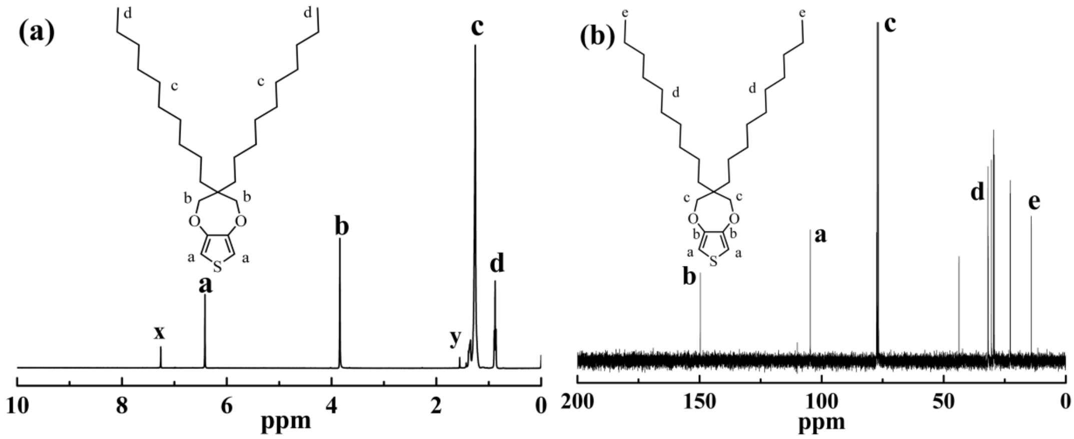

Figure. S2. ^{1}H NMR (a) and ^{13}C NMR (b) spectra of 3,3-didecyl-3,4-dihydro-2H-thieno[3,4-b][1,4]dioxepine, ‘x’ represents the $CDCl_3$ solvent peak and ‘y’ represents the water peak.

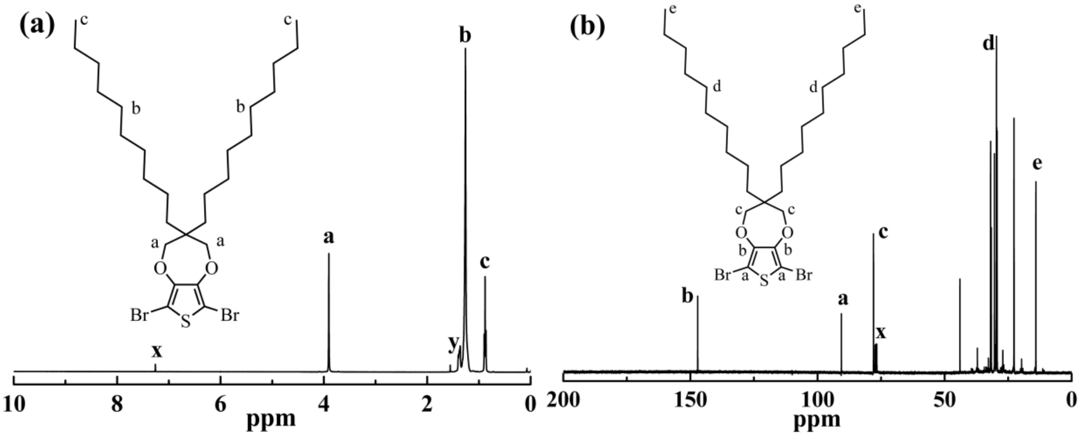

Figure. S3. ^{1}H NMR (a) and ^{13}C NMR (b) spectra of 6,8-dibromo-3,3-didecyl-3,4-dihydro-2H-thieno[3,4-b][1,4]dioxepine, ‘x’ represents the $CDCl_3$ solvent peak and ‘y’ represents the water peak.

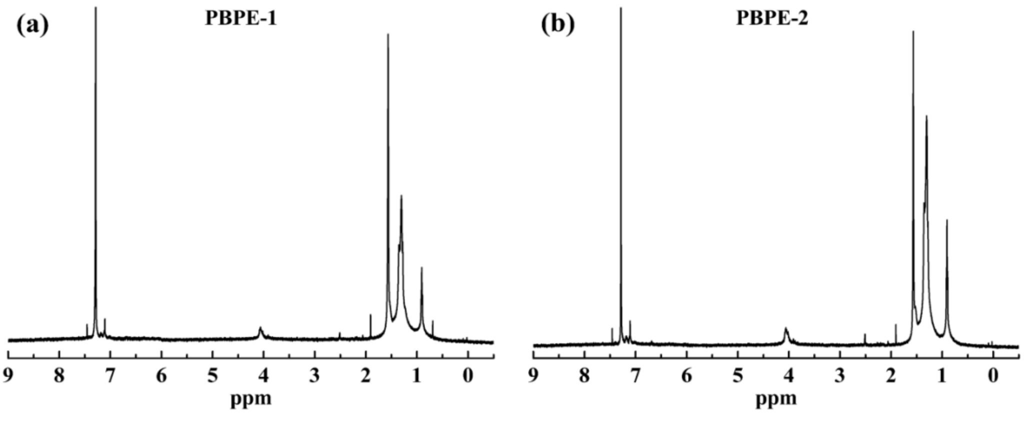

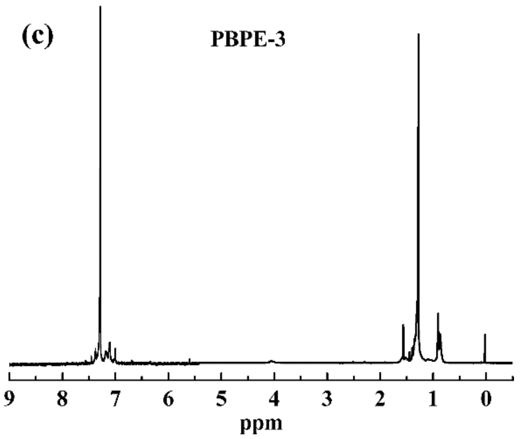

Figure. S4. ^{1}H NMR spectra of PTPE-1(a), PTPE-2(b), PTPE-3(c)

3. XPS spectra of the polymers

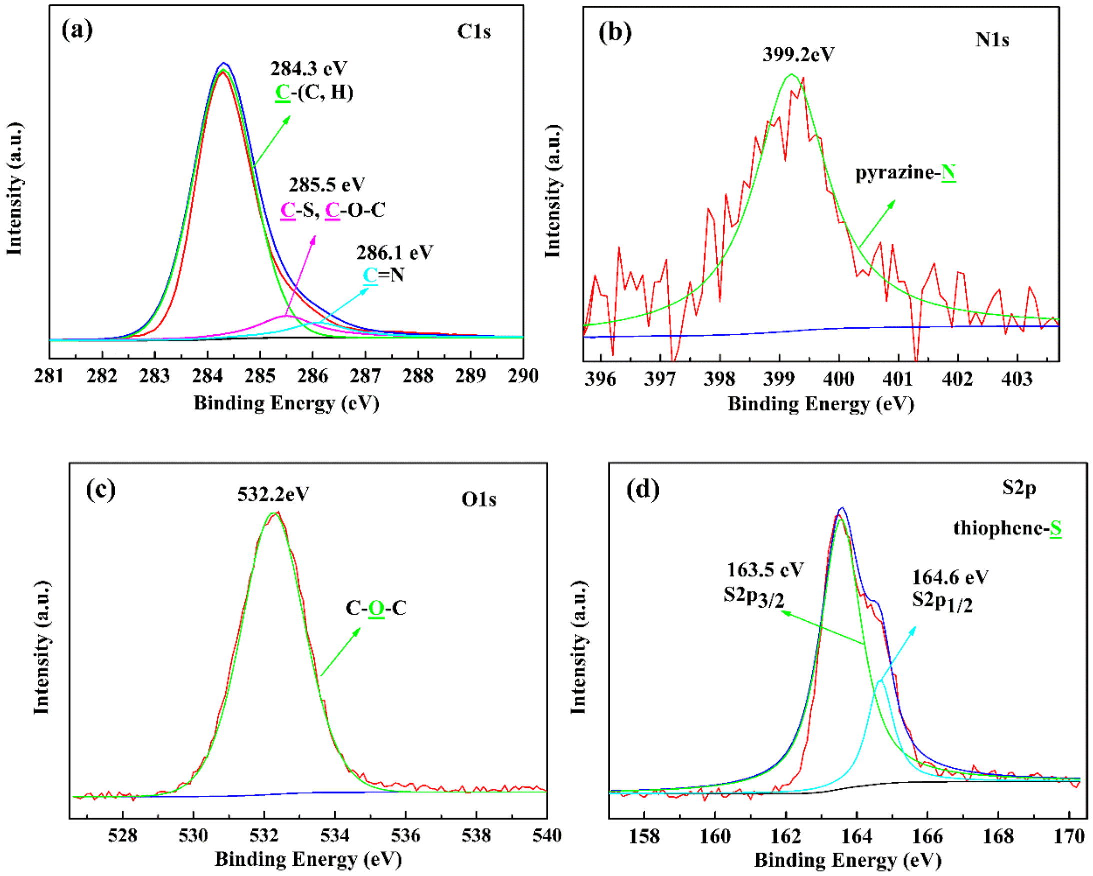

Figure. S5. XPS spectra of polymer PBPE-2: (a) C1s, (b) N1s, (c) O1s and (d) S2p.

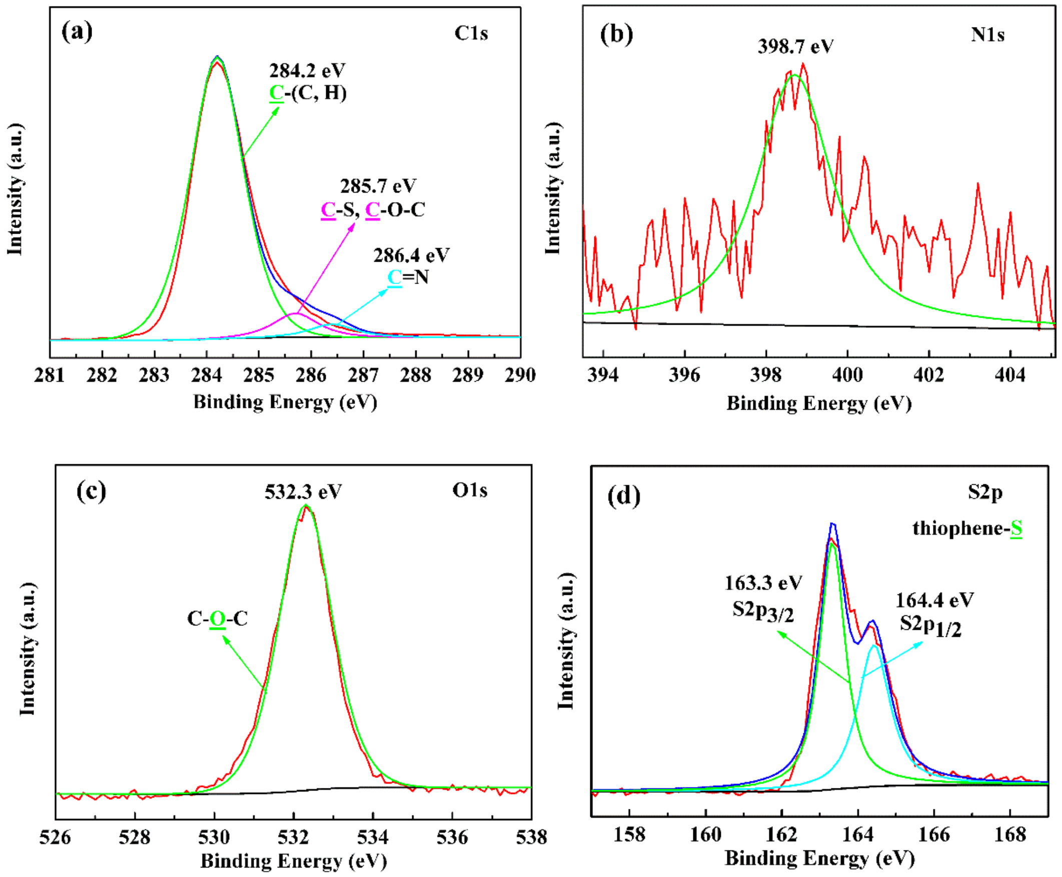

Figure. S6. XPS spectra of polymer PBPE-3: (a) C1s, (b) N1s, (c) O1s and (d) S2p.

4. Electrochromic switching studies

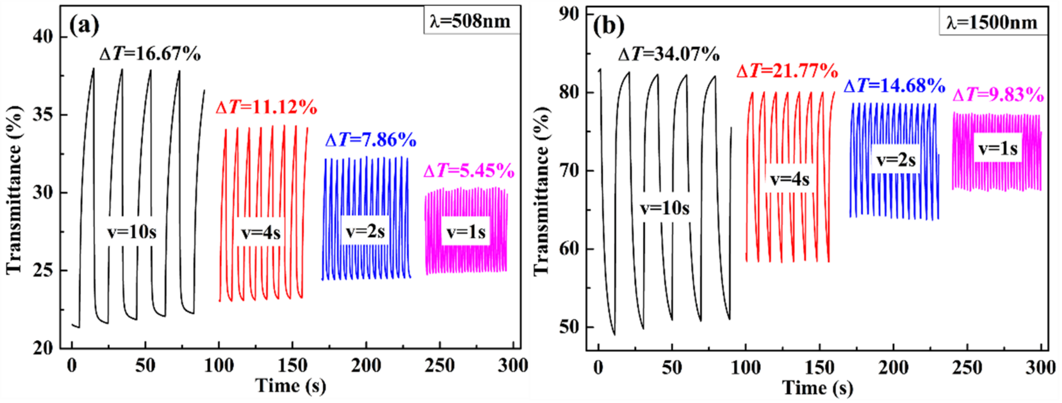

Figure. S7. Electrochromic switching of PBPE-2 at (a) 508nm and (b) 1500 nm with intervals of 10 s, 4 s, 2 s and 1 s.

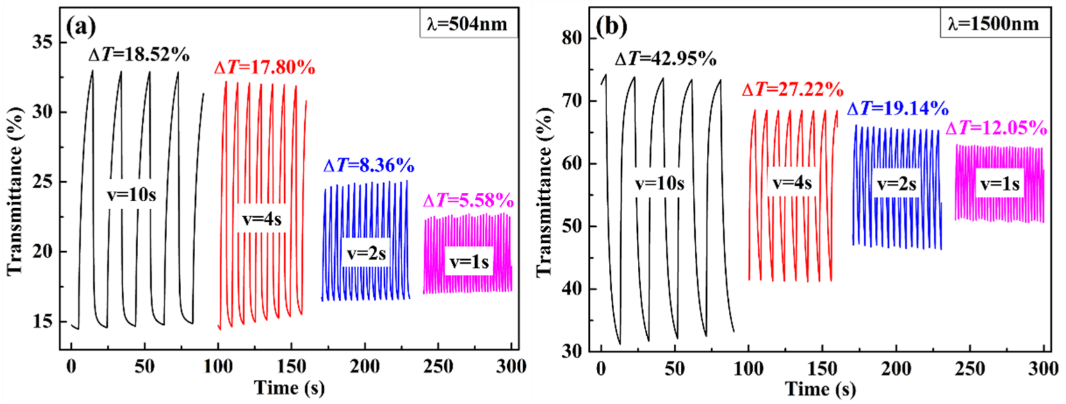

Figure. S8. Electrochromic switching of PBPE-3 at (a) 504 nm and (b) 1500 nm with intervals of 10 s, 4 s, 2 s and 1 s.

5. Colorimetry

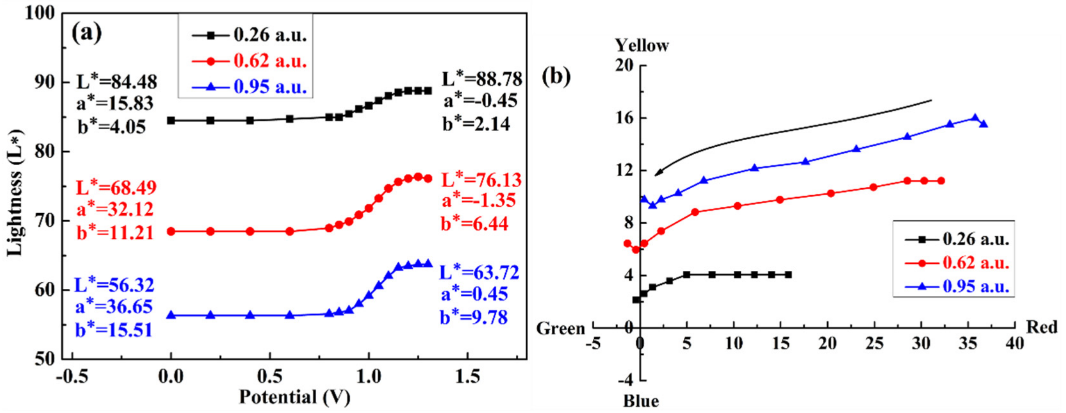

Figure. S9. (a) Lightness (L*) and (b) colorimetry values (a* b*) for the PBPE-2 films in three different optical densities as a function of applied potential.

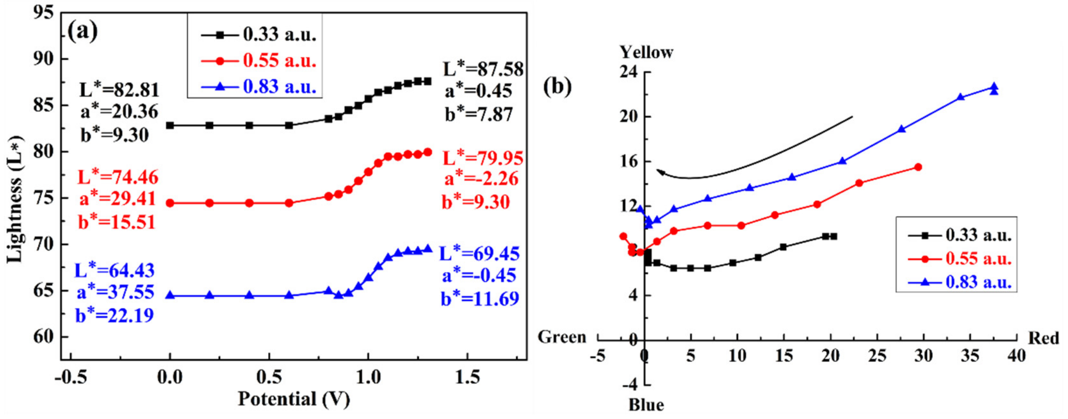

Figure. S10. (a) Lightness (L*) and (b) colorimetry values (a* b*) for the PBPE-3 films in three different optical densities as a function of applied potential.
